# The endothelial-immunothrombotic storm in viral sepsis: lessons from COVID-19

**DOI:** 10.3389/fimmu.2025.1681764

**Published:** 2026-01-06

**Authors:** Kaihuan Zhou, Yin Chen, Jielong Pang, Jianfeng Zhang, Junyu Lu

**Affiliations:** 1 Intensive Care Unit, The Second Affiliated Hospital of Guangxi Medical University, Nanning, China; 2 Intensive Care Unit, The First Affiliated Hospital of Guangxi Medical University, Nanning, China; 3 Department of Emergency Medicine, The Second Affiliated Hospital of Guangxi Medical University, Nanning, China; 4 Department of Emergency Medicine, Wuming Hospital of Guangxi Medical University, Nanning, China

**Keywords:** COVID-19, cytokine storm, endothelial injury, immunothrombosis, viral sepsis

## Abstract

Taking COVID-19 as an illustrative example, this review systematically elucidates the central pathological mechanism of viral sepsis, termed the endothelial-immunothrombotic storm. This mechanism is initiated by the direct viral infection of endothelial cells, which provokes excessive immune activation and disrupts coagulation through immunothrombosis, including cytokine storms, NETosis, and complement activation. Meanwhile, these processes establish a vicious cycle leading to multiple organ failure. Compared with classical bacterial sepsis, viral sepsis exhibits distinctive features such as interferon dysregulation, direct endothelial damage, a hypercoagulable state, and T-cell exhaustion. This review integrates the latest research findings, contrasts the pathophysiological differences between viral and bacterial sepsis, and proposes precision strategies focused on endothelial protection, immune modulation, and anticoagulation. Finally, we discuss the clinical translational prospects of these approaches and suggests directions for future research.

## 1 Introduction

Sepsis is a life-threatening organ dysfunction caused by a dysregulated host response to infection and remains one of the leading causes of morbidity and mortality worldwide, imposing a substantial burden on healthcare systems (1, 2). As a highly heterogeneous syndrome triggered by diverse pathogens, its etiological spectrum extends beyond bacteria to include viruses and fungi (2). Although bacterial infections are most common, the contribution of viral pathogens to sepsis has long been underestimated. Notably, up to 42% of patients with sepsis have negative microbiological results, suggesting that a considerable proportion may arise from non-bacterial causes (3, 4).

The pandemic caused by severe acute respiratory syndrome coronavirus 2 (SARS-CoV-2), which results in Coronavirus Disease 2019 (COVID-19), has revealed a series of distinctive pathological features underlying virus-induced organ dysfunction and has provided critical evidence for re-examining the core mechanisms of viral sepsis (5). Patients with severe COVID-19 frequently develop acute respiratory failure, diffuse microvascular injury and multiple organ dysfunction syndrome (MODS), manifestations that closely align with the Sepsis-3 definition of sepsis (1, 6). These clinical and pathological features primarily arose from an uncontrolled host immune response to viral infection, resulting in systemic inflammation and organ injury (7). Accumulating evidence further shows that endothelial injury, excessive immune activation and dysregulated coagulation form a self-amplifying loop, in which endothelial dysfunction, immune dysregulation and immunothrombosis are tightly interconnected. This pathological process, referred to as the endothelial-immunothrombotic storm, is considered a key driver of disease progression, MODS and death in COVID-19 (6, 8, 9).

Despite the extensive literature on COVID-19 and sepsis, most existing reviews discuss endothelial injury, immune dysregulation or coagulopathy as separate entities, without providing an integrated mechanistic framework that captures their reciprocal reinforcement. Moreover, few studies have systematically compared viral and bacterial sepsis from a mechanistic pathway perspective, particularly regarding endothelial vulnerability, immune signatures and coagulation phenotypes. Accordingly, a synthesis that bridges pathogen categories and integrates the endothelial, immune and coagulation axes is still urgently needed to advance conceptual understanding and support clinical translation in this field.

Against this background, this review employs COVID-19 as an illustrative model to refine the concept of the endothelial-immunothrombotic storm, providing an integrated explanation of how endothelial injury, immune dysregulation and immunothrombosis reciprocally reinforce one another. Building on this framework, we systematically compare the mechanistic features of multiple forms of viral sepsis and delineate their distinctions from bacterial sepsis. We further examine the clinical and translational implications along the continuum of mechanisms–biomarkers–therapeutic strategies, with the aim of offering a consolidated perspective to support precision diagnosis and targeted interventions for viral sepsis.

## 2 Early mechanisms of viral sepsis: from pathogen recognition to initial signaling

### 2.1 Pathogen recognition and initiation of innate immunity: the divergence between bacterial and viral sepsis

Sepsis begins with the host’s recognition of invading pathogens, yet viruses and bacteria engage fundamentally distinct immunological entry points. Bacterial sepsis is typically initiated by bacterial toxins and cell-wall components, such as lipopolysaccharide, lipoteichoic acid and bacterial DNA, which function as pathogen-associated molecular patterns (PAMPs). These ligands primarily activate Toll-like receptor (TLR) 2 and TLR4 on the cell membrane, triggering a high-amplitude pro-inflammatory cascade centered on nuclear factor κB (NF-κB) (10). This recognition pattern is characterized by a locally initiated response dominated by a single immune-cell lineage, with inflammatory signals spreading outward from focal sites. Consequently, the early response is intense yet anatomically and functionally more confined.

In contrast, the initiation of viral sepsis relies on intracellular sensing of viral replicative nucleic acids (11). Double-stranded RNA, single-stranded RNA and aberrant DNA generated during viral replication are detected by nucleic acid sensors such as TLR3/7/8/9, retinoic acid-inducible gene I (RIG-I), melanoma differentiation-associated gene 5 (MDA5) and the cyclic GMP–AMP synthase–stimulator of interferon genes (cGAS–STING) pathway. These receptors are expressed not only in immune cells but also in epithelial, endothelial and parenchymal cells, resulting in a synchronized, cross-lineage mode of pathogen recognition (11–14).

This broad sensing architecture leads to rapid activation of the interferon network and widespread induction of chemokines such as CXCL8 and CXCL10, effectively recruiting non-immune cells into the inflammatory milieu (14). Unlike the focal, lineage-centered initiation typical of bacterial sepsis, viral infection establishes a multi-node, parallel inflammatory topology from the outset, creating a distinct biological foundation for the subsequent escalation of endothelial activation, immune dysregulation and focal immunothrombosis (6, 11, 14).

### 2.2 Host response specificity in viral sepsis

The early host response in viral sepsis exhibits pronounced temporal dependence, governed primarily by the interferon network activated through intracellular nucleic acid sensors (15). Viral nucleic acids detected by TLR3/7/8/9, RIG-I, MDA5 and the cGAS–STING pathway initiate type I and type III interferon signaling, but this response must occur within an appropriate temporal window to effectively restrain viral replication (14, 16). Insufficient or delayed interferon production in the early phase allows accelerated viral propagation and amplifies subsequent inflammatory stress (17). Conversely, a sustained or excessive interferon response drives overexpression of chemokines such as CXCL10 and CCL2, promoting broader propagation of inflammation (18). Thus, interferon signaling in viral infection exerts both antiviral and immunopathogenic effects, creating an inherently unstable foundation for the early host response in viral sepsis (19).

In addition, many viruses employ immune-evasion strategies that disrupt the host antiviral machinery, including suppression of interferon cascades, attenuation of antigen presentation and inhibition of the Janus kinase–signal transducer and activator of transcription (JAK–STAT) pathway (20–22). These mechanisms hinder effective viral clearance during the early phase of infection, leaving the host in a prolonged state where inflammatory drivers and immunosuppressive forces coexist. Such a response pattern not only prolongs viral replication but also lowers the tolerance threshold of non-immune cells, particularly endothelial cells, to inflammatory stimuli, thereby predisposing them to later systemic inflammatory and coagulopathic disturbances (6, 22, 23). Collectively, these host-response features establish a critical biological basis for the ensuing endothelial injury and immunothrombotic manifestations that typify viral sepsis.

### 2.3 COVID-19: a catalyst for advancing understanding of viral sepsis

The COVID-19 pandemic has generated an extensive body of clinical, pathological and multi-omics evidence, offering a critical window into the systems-biology features of viral sepsis (6, 24, 25). Research centered on SARS-CoV-2 demonstrates that viral invasion activates multiple injurious pathways simultaneously: endothelial cells emerge as one of the earliest and most central targets; the immune response progressively shifts from antiviral defense to immunopathology, marked by pronounced cytokine elevation and profound lymphopenia (26); and the coagulation–fibrinolytic system develops highly distinctive abnormalities accompanied by widespread microthrombosis (6, 27). These insights have not only advanced understanding of COVID-19 pathogenesis but have also catalyzed broader recognition of the shared pathological foundations of viral sepsis, enabling a more integrated delineation of the interplay between virus-induced endothelial injury, immune dysregulation and coagulopathic disturbances.

## 3 Core mechanisms of the endothelial-immunothrombotic storm

The endothelial-immunothrombotic storm refers to a self-perpetuating pathological cycle seen in viral sepsis, particularly in COVID-19, in which viral invasion triggers a host response integrating three mutually reinforcing processes: endothelial cell injury, excessive and dysregulated immune activation, and aberrant activation of the coagulation system leading to immunothrombosis. Together, these interconnected pathways drive progression toward MODS (28–30).

### 3.1 Systemic expansion of immune activation: a cross-cellular synchronous response

Following viral invasion, innate immune sensing through TLR3/7/8/9, RIG-I and MDA5 rapidly activates the IRF3/7 and NF-κB axes, leading to robust induction of type I/III IFNs and chemokines such as CXCL8, CXCL10 and CCL2 (14, 31). As these signals propagate across tissue compartments and diversify among immune-cell subsets, the response transitions into a synchronized, cross-lineage activation program. This dynamic amplification network encompasses dendritic cells, macrophages, neutrophils and extends to epithelial and endothelial compartments (32, 33).

Chemokine-driven macrophage recruitment promotes M1 polarization, accompanied by high-level release of IL-6, TNF-α and IL-1β, which further intensify the inflammatory milieu (34). Neutrophils, mobilized via CCL2-dependent chemotaxis, accumulate at sites of infection and become primed for subsequent neutrophil extracellular trap formation (NETosis) (35). Endothelial cells, simultaneously exposed to IFNs, inflammatory mediators and viral ligands, rapidly acquire a pro-inflammatory and pro-coagulant phenotype, effectively functioning as an early amplification hub for both inflammatory and hemostatic signaling (6, 16, 36). By this stage, a local antiviral response has evolved into a systemic immune reprogramming process. Without adequate regulatory containment, this synchronized network drives the characteristic immune–endothelial–coagulatory interplay of viral sepsis and establishes a pivotal upstream axis leading to MODS (6).

### 3.2 The endothelium as a central hub for inflammatory and coagulatory signaling

The endothelium serves as a critical interface linking the immune and coagulation systems and, in viral sepsis, rapidly transitions from a structural barrier to a central node for integrating and amplifying pathological signals (29). A range of endotheliotropic viruses, including influenza virus, dengue virus, hantaviruses and SARS-CoV-2, can directly infect endothelial cells, causing primary injury characterized by cytoskeletal disruption, disassembly of adhesion structures and programmed cell death (37, 38). Viral proteins and replicative intermediates additionally activate endothelial innate-sensing pathways, further intensifying damage-associated signaling (39). Collectively, these upstream events precipitate a rapid transition of the endothelium into an activated, proinflammatory, and procoagulant state.

Systemic inflammatory mediators, along with reactive oxygen species (ROS), the complement terminal complex (C5b-9), and various cytotoxic effectors, induce additional endothelial injury through extrinsic pathways. These insults disrupt tight junctions, degrade the glycocalyx, and downregulate anticoagulant molecules, further remodeling the endothelial phenotype toward a strongly proinflammatory and prothrombotic state (36, 40). Once activated, endothelial cells up-regulate adhesion molecules such as intercellular adhesion molecule-1(ICAM-1), vascular cell adhesion molecule-1 (VCAM-1) and E-selectin, thereby promoting leukocyte adhesion and trans-endothelial migration. Concurrently, they release Willebrand factor (vWF), increase tissue factor expression and elevate fibrinolytic inhibitors, collectively driving local and ultimately systemic immunothrombosis (41).

At the microcirculatory level, endothelial activation becomes widespread, characterized by endotheliitis, basement membrane exposure, and microthrombus deposition. Autopsy studies in patients with COVID-19 have further demonstrated extensive endothelial injury and *in situ* thrombosis, supporting the view that endothelial dysfunction is a primary driver of the shift from a localized antiviral response to systemic pathology in viral sepsis (6, 42). Overall, the endothelium acts as a central platform where inflammatory, complement, and coagulation signals converge, and its pathological activation plays a critical role in the progression of viral sepsis and the development of MODS.

### 3.3 Biphasic immune deviation: synergistic progression of pro-inflammatory amplification and immune exhaustion

In the early phase, viral nucleic acids activated innate immune pathways through PRRs, driving macrophage polarization toward the M1 phenotype and triggering the abundant release of IL-6, TNF-α, and IL-1β, which rapidly amplified the inflammatory cascade (34). Concurrently, neutrophils recruited by C-X-C motif CXCL8 and CCL2 underwent enhanced NETosis, and the resulting neutrophil extracellular traps (NETs) exerted marked endothelial toxicity and initiated the coagulation cascade, thereby promoting immunothrombosis (9, 43). In addition, complement activation through multiple pathways generated C3a, C5a, and C5b-9, which exerted pro-inflammatory, chemotactic, and pro-coagulant effects, enabling the immune–endothelial–coagulation interplay to expand rapidly from local to systemic levels (44, 45).

However, when the immune response deviates from proper regulation, antiviral defense transitions into a state characterized by the parallel evolution of a persistently pro-inflammatory innate immune response and a progressively declining adaptive immune response. This leads to escalating inflammation coupled with diminished clearance capacity, thereby constituting a key driver of immune imbalance in viral sepsis. Observational and multi-omics studies have consistently demonstrated that critically ill COVID-19 patients commonly exhibit profound peripheral lymphopenia (46–48). Notably, CD4^+^ and CD8^+^ T cells display a marked exhaustion phenotype, evidenced by the sustained upregulation of inhibitory receptors such as programmed cell death protein 1 (PD-1), T cell immunoglobulin and mucin domain-containing protein 3 (TIM-3), and LAG-3, mitochondrial bioenergetic dysfunction, suppressed T-cell receptor (TCR) signaling, and an epigenetically programmed exhaustion landscape (46–48). Concurrently, clinical studies confirm that during the severe stage of illness, Th1 and Th17 responses become abnormally amplified, while the number and function of regulatory T cells are synchronously reduced. This imbalance in the effector-regulatory axis compromises the maintenance of virus-specific immunity and further exacerbates immunopathological inflammation (49, 50).

Furthermore, the B-cell compartment exhibits phenotypic dysregulation. In some patients, abnormal polyclonal activation and the production of autoantibodies (e.g., anti-phospholipid and anti-type I interferon antibodies) are observed. These autoantibodies not only weaken antiviral defenses but may also directly contribute to pathological processes such as vasculitis and coagulopathy (51, 52). Concurrently, viral sepsis is accompanied by functional decline in innate immune effectors, with the synergistic dysfunction of NK cells and myeloid-derived suppressor cells (MDSCs) being particularly prominent. In the setting of persistent inflammation and inhibitory cytokines, NK cells undergo numerical reduction and profound exhaustion. This is characterized by diminished degranulation activity, reduced IFN-γ production, and downregulation of activating receptors (e.g., NKG2D), thereby impairing the rapid clearance of virus-infected cells and compromising early immune braking (53, 54). Similarly, paralleling the decline in NK cell cytotoxicity, inflammation-driven expansion of MDSCs occurs. These cells release ROS, arginase-1, and IL-10, which suppress T-cell proliferation, disrupt antigen presentation, and limit cytotoxic responses. This further consolidates the immune exhaustion phenotype and elevates the risk of secondary infections (55, 56). The mutual amplification between these two cell populations drives both innate and adaptive immunity into a state of profound suppression, creating a biphasic imbalance characterized by persistent inflammation coexisting with immune exhaustion. This process propels viral sepsis from a defensive response toward immunopathology.

### 3.4 Immunomediated coagulation activation and microthrombosis

Immunothrombosis refers to a host defense mechanism in which the innate immune system and the coagulation cascade are intricately coupled within the microcirculation to limit pathogen dissemination. In sepsis, particularly viral sepsis, this physiological response becomes abnormally amplified and dysregulated, evolving into a key pathological process that drives organ injury (29).

Firstly, both the extrinsic and intrinsic coagulation pathways are abnormally activated. Damaged endothelium, activated monocytes/macrophages, and NETs can all express high levels of tissue factor, constantly triggering the extrinsic pathway (30, 36). Simultaneously, NETs, which are rich in negatively charged DNA and histones, provide a potent contact activation surface for coagulation factor XII (FXII), thereby initiating the intrinsic coagulation cascade and the associated kallikrein-kinin system (KKS) (57, 58). Proteomic studies have demonstrated that impaired NET clearance sustains FXII activation, exacerbating the coagulation-inflammation cycle within the lungs of COVID-19 patients. This evidence suggests that the NET-FXII axis serves as a critical amplifier of viral immunothrombosis (57).

Platelets play a crucial effector role in inflammation-coagulation coupling. Multiple viruses, including SARS-CoV-2, can directly induce platelet activation. Inflammatory mediators (e.g., platelet-activating factor, thromboxane A_2_), elevated von vWF, and NET components can further promote platelet aggregation and degranulation. This releases procoagulant factors such as adenosine diphosphate and thrombin, leading to the formation of platelet-rich microthrombi (59). Furthermore, adhesive complexes formed between platelets and neutrophils can drive additional NET generation and amplify the activation of both the complement and coagulation pathways, establishing a characteristic immunocoagulation positive feedback loop (60).

Simultaneously, the natural anticoagulant and fibrinolytic functions of the endothelium are significantly impaired. Inflammation leads to the downregulation of thrombomodulin, endothelial protein C receptor, tissue factor pathway inhibitor, and heparin-like glycosaminoglycans. This attenuation weakens the anticoagulant and anti-inflammatory actions of the protein C system (61, 62). Regarding the fibrinolytic system, plasminogen activator inhibitor-1 (PAI-1) is commonly elevated in both sepsis and COVID-19. This potent increase strongly inhibits the activity of urokinase-type and tissue-type plasminogen activators (uPA and tPA), thereby obstructing the degradation of formed fibrin networks and resulting in more stable and persistent thrombi (63, 64).

Among these mechanisms, NETs serve as the central structural and functional hub. Their DNA-protein meshwork not only provides a three-dimensional scaffold for platelets, red blood cells, and coagulation factors, promoting thrombus deposition, but also directly damages the endothelium and induces a procoagulant phenotype via histones, elastase, and myeloperoxidase (36, 65). Furthermore, a positive feedback loop exists between NETs and the complement system, continuously amplifying coagulation-inflammation signaling. When NET clearance is impaired (e.g., due to reduced DNase activity) and leads to their persistent accumulation, sustained activation of FXII is maintained. This drives the progression from focal microthrombosis to systemic coagulation imbalance (57, 66). Consequently, excessive NET formation coupled with impaired clearance is a key driver that transforms immunothrombosis from a defensive response into a pathologically amplified state.

Ultimately, the sustained activation of both coagulation pathways, the amplified interactions between platelets, leukocytes, and the endothelium, and the comprehensive suppression of anticoagulant and fibrinolytic systems collectively lead to widespread microvascular thrombosis in organs such as the lungs, kidneys, myocardium, and brain. These thrombi significantly obstruct microcirculatory perfusion, causing localized hypoperfusion and tissue hypoxia. This represents a critical terminal event in the progression of sepsis toward MODS (6, 41).

### 3.5 The vicious cycle of the endothelial-immunothrombotic storm

**Figure 1:**
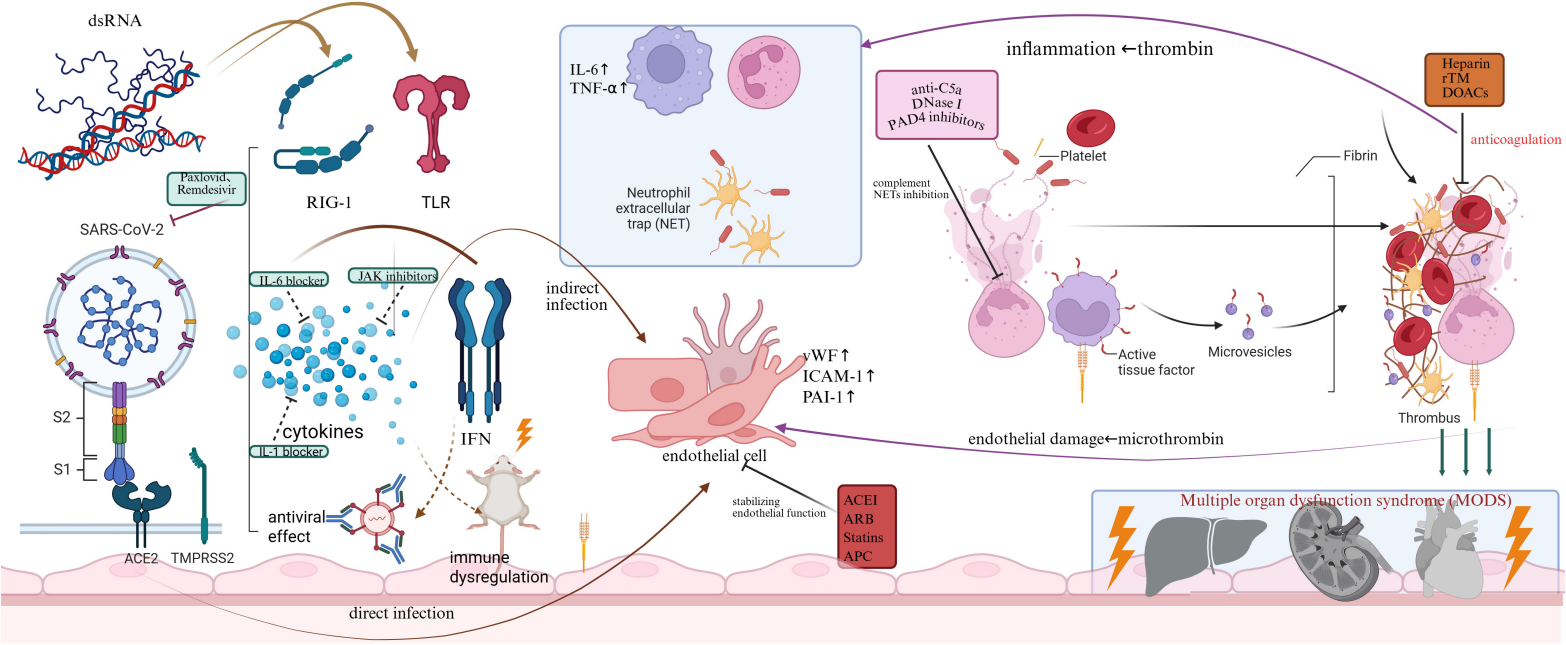
Conceptual diagram of the Endothelial-Immunothrombotic Storm mechanism in viral sepsis and potential multi-target therapeutic interventions. SARS-CoV-2 enters endothelial cells via ACE2 receptors, releasing PAMPs that are sensed by PRRs such as TLRs and RIG-I. This recognition activates downstream signaling and induces IFN-I/III and proinflammatory cytokines (e.g., IL-6, TNF-α), contributing to antiviral defense but also promoting immune dysregulation. Excessive cytokine release activates neutrophils, induces NET formation, and amplifies the activation of monocytes, platelets, and the complement cascade, collectively engaging TF-related pathways and microvesicle release, ultimately driving fibrin deposition and thrombosis. Meanwhile, SARS-CoV-2 directly injures endothelial cells or indirectly triggers proinflammatory and procoagulant endothelial phenotypes (increased ICAM-1, vWF, PAI-1) via inflammatory mediators, disrupting the vascular barrier and accelerating a prothrombotic state. The interplay between immune hyperactivation, endothelial dysfunction, and coagulation disturbances constitutes a positive-feedback loop described as the endothelial-immunothrombotic storm, driving microvascular thrombosis, tissue ischemia, hypoxia, and progression to MODS. This figure also integrates multi-target therapeutic interventions, including antiviral agents (Paxlovid, Remdesivir); immunomodulatory therapies (IL-6/IL-1 antagonists, JAK inhibitors, Treg-enhancing strategies); anticoagulants (heparin, rTM, DOACs); endothelial-protective agents (ACE inhibitors, ARBs, statins, APC); and complement/NET-directed therapies (anti-C5a, DNase I, PAD4 inhibitors), collectively illustrating a multidimensional, synergistic treatment concept for viral sepsis. ACE2, angiotensin-converting enzyme 2; APC, activated protein C; ARB, angiotensin receptor blocker; C5a, complement component 5a; DOAC, direct oral anticoagulant; DNase, deoxyribonuclease; ICAM-1, intercellular adhesion molecule-1; IFN, interferon; IL, interleukin; JAK, Janus kinase; MODS, multiple organ dysfunction syndrome; NET, neutrophil extracellular trap; PAD4, peptidylarginine deiminase 4; PAI-1, plasminogen activator inhibitor-1; PAMP, pathogen-associated molecular pattern; PRR, pattern recognition receptor; rTM, recombinant thrombomodulin; RIG-I, retinoic acid–inducible gene I; SARS-CoV-2, severe acute respiratory syndrome coronavirus 2; TF, tissue factor; TLR, Toll-like receptor; TNF-α, tumor necrosis factor-alpha; Treg, regulatory T cell; vWF, von Willebrand factor.

In summary, the three core pathological processes outlined above, endothelial injury, immune dysregulation, and coagulation activation, do not occur in isolation. Instead, they form a mutually reinforcing and progressively amplifying vicious cycle, collectively constituting the complete pathophysiological picture of the endothelial-immunothrombotic storm (**Figure 1**). This cycle is initiated by virus-triggered innate immunity and concurrent endothelial damage. The subsequently activated endothelium recruits and further activates immune cells, thereby driving immunothrombosis. In turn, the aberrant activation of coagulation pathways reciprocally exacerbates both endothelial and immune dysfunction. Throughout this cycle, key nodal molecules and effectors, such as NETs, complement factors, and pivotal cytokines, serve as critical amplifiers and connectors. The resultant microcirculatory dysfunction, causing tissue hypoxia and metabolic derangement, further entrenches this positive feedback loop. This relentless cycle propels the pathological cascade from a local to a systemic scale and from a potentially controllable state to one of dysregulated escalation, ultimately surpassing the body’s compensatory capacity and culminating in irreversible multiple organ dysfunction.

## 4 Similarities and differences in the “storm” between COVID-19 and classical bacterial sepsis

**Figure 2:**
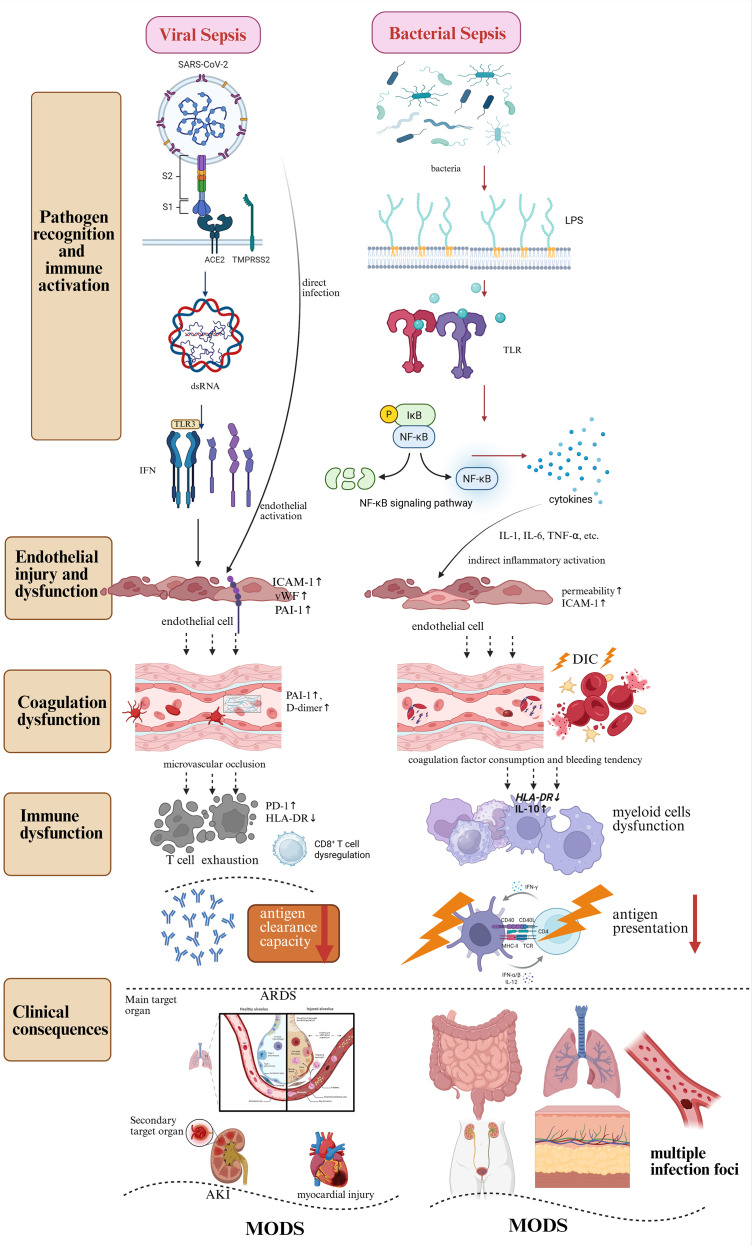
Comparative mechanistic overview of viral versus bacterial sepsis across pathogen recognition, immune activation, endothelial dysfunction, coagulation disturbances, immune dysregulation, and clinical outcomes. Viral sepsis, exemplified by SARS-CoV-2 infection, involves direct endothelial invasion via ACE2 receptors and recognition of viral PAMPs such as double-stranded RNA through PRRs including TLRs (3/7/9) and RIG-I. This initiates IFN-I/III production and a spectrum of proinflammatory cytokines, contributing to antiviral defense but also maladaptive immune activation. In contrast, bacterial sepsis predominantly activates TLR4/TLR2 and NOD pathways in response to PAMPs such as LPS and peptidoglycan, triggering NF-κB–mediated inflammatory cascades and cytokine storm. Regarding endothelial dysfunction, viruses can directly induce endothelial apoptosis or promote a proinflammatory/procoagulant phenotype (increased ICAM-1, vWF, PAI-1), facilitating microvascular thrombosis. Bacterial pathogens primarily provoke secondary endothelial activation through systemic inflammatory mediators (e.g., IL-1, TNF-α), leading to barrier disruption, enhanced permeability, and adhesion molecule upregulation. With respect to coagulation disturbances, viral sepsis is characterized by fibrinolysis suppression with elevated PAI-1 levels, predisposing to microvascular thrombosis and marked D-dimer elevation. In contrast, bacterial sepsis more often progresses to DIC with consumptive coagulopathy and bleeding tendency. At the level of immune dysregulation, viral sepsis is associated with T-cell exhaustion (PD-1 upregulation, HLA-DR downregulation), CD8^+^ T-cell dysfunction, and potential autoantibody production. Bacterial sepsis is more commonly linked to myeloid dysfunction, impaired antigen presentation, and profound immunosuppression alternating with hyperinflammation. Clinically, viral sepsis typically manifests with ARDS and pulmonary microthrombosis as predominant features, with secondary renal and cardiac microcirculatory involvement, progressing to MODS. In contrast, bacterial sepsis exhibits heterogeneous infection foci—such as intra-abdominal, urinary tract, or soft-tissue infections—often complicated by DIC and multiorgan failure, with outcomes strongly influenced by timely source control and organ support. ACE2, angiotensin-converting enzyme 2; ARDS, acute respiratory distress syndrome; DIC, disseminated intravascular coagulation; HLA-DR, human leukocyte antigen–DR; ICAM-1, intercellular adhesion molecule-1; IL, interleukin; LPS, lipopolysaccharide; MODS, multiple organ dysfunction syndrome; NF-κB, nuclear factor kappa-light-chain-enhancer of activated B cells; NOD, nucleotide-binding oligomerization domain; PAI-1, plasminogen activator inhibitor-1; PAMP, pathogen-associated molecular pattern; PD-1, programmed cell death protein-1; PRR, pattern recognition receptor; RIG-I, retinoic acid–inducible gene I; SARS-CoV-2, severe acute respiratory syndrome coronavirus 2; TLR, Toll-like receptor; TNF-α, tumor necrosis factor-alpha; vWF, von Willebrand factor.

Viral sepsis, as exemplified by COVID-19, and classical bacterial sepsis may ultimately converge on similar terminal pathological phenotypes, including systemic inflammation, widespread endothelial dysfunction, immunothrombosis, and MODS. However, they differ significantly in the initial triggers of the storm, the specific immune-endothelial-coagulation interplay pathways, the patterns of microcirculatory involvement, and the programs of immune exhaustion (**Table 1**, **Figure 2**).

**Table 1:** Comparison of pathophysiology, clinical features, and treatment strategies between viral and bacterial sepsis.

Dimension	Viral sepsis (e.g., COVID-19)	Bacterial sepsis	Key differences	Reference(s)
Pathogen Triggers	• Viral PAMPs: dsRNA/ssRNA (TLR3/7/8, RIG-I) • Direct endothelial infection via receptors such as ACE2 (SARS-CoV-2)	• Bacterial PAMPs: LPS (TLR4), peptidoglycan (TLR2) • Indirect endothelial injury via circulating toxins	Viruses can directly invade the endothelium, initiating primary structural damage	(10–14)
Immune Response	• Early robust IFN response: double-edged sword (antiviral vs. immunopathology) • Profound T-cell exhaustion: CD4+/CD8+↓, PD-1↑ • Autoantibodies: anti-IFN, antiphospholipid antibodies	• TLR/NF-κB–driven: TNF-α, IL-1β, IL-6 surge • Myeloid cell activation: monocyte/macrophage dominated	Virus-specific contradictory IFN effects & autoimmunity	(15, 16, 70)
Endothelial Injury	• Endotheliitis: widespread microvascular injury, basement membrane exposure • ACE2 downregulation: RAAS imbalance → vasoconstriction ↑	• Secondary activation: increased permeability induced by inflammatory mediators (TNF-α) • Glycocalyx shedding: barrier dysfunction	Viruses cause direct structural injury, bacteria primarily functional dysregulation	(81, 82, 93)
Coagulation Dysfunction	• Hypercoagulable state: markedly elevated D-dimer, fibrinogen ↑, frequent microthrombosis • Fibrinolysis inhibition: PAI-1 markedly elevated • NETs-driven: strong procoagulant activity of histones	• Consumptive DIC: platelet ↓, prolonged PT/aPTT, increased bleeding risk • Coexistence of microthrombosis and bleeding	Viral hypercoagulability is prominent, bacterial sepsis has higher bleeding risk	(72, 73, 77, 78)
Organ Involvement Patterns	• Lungs as the core target: ARDS, *in situ* pulmonary thrombosis • Acute kidney injury: driven by microthrombosis and endotheliitis	• Multifocal infection: primary sites such as abdominal or urinary tract • Early-onset shock: decreased cardiac output, reduced peripheral resistance	Viruses primarily target the respiratory system, bacterial infections have diverse foci	(29, 70, 99–101)
Differences in Treatment Strategies	• Antivirals: Paxlovid/remdesivir (early phase) • Anticoagulation: therapeutic-dose heparin (prevent microthrombosis) • Immunomodulation: JAK inhibitors/IFN modulation	• Antibiotics: broad-spectrum coverage (golden hour) • Anticoagulation: prophylactic-dose heparin (prevent DIC) • Immune enhancement: GM-CSF (counteract immune paralysis)	Viral sepsis requires targeting viral replication + immunomodulation, bacterial sepsis needs rapid pathogen clearance	(119, 123–130)

sTM, soluble thrombomodulin; Ang-2, angiopoietin-2; ICAM-1, intercellular adhesion molecule-1; VCAM-1, vascular cell adhesion molecule-1; vWF, von Willebrand factor; ADAMTS13, a disintegrin and metalloproteinase with thrombospondin motifs 13; IL-6, interleukin-6; IL-1RA, interleukin-1 receptor antagonist; TNF-α, tumor necrosis factor-alpha; IL-8, interleukin-8; CXCL10 (IP-10), C-X-C motif chemokine ligand 10; sCD14, soluble CD14; sCD163, soluble CD163; HLA-DR, human leukocyte antigen–DR; PD-1, programmed cell death protein 1; TIM-3, T-cell immunoglobulin and mucin-domain containing-3; LAG-3, lymphocyte activation gene 3; TAT, thrombin–antithrombin complex; F1 + 2, prothrombin fragment 1 + 2; PAI-1, plasminogen activator inhibitor-1; cfDNA, cell-free DNA; MPO-DNA, myeloperoxidase–DNA complex; CitH3, citrullinated histone H3; C3a and C5a, complement activation products; sC5b-9, soluble terminal complement complex; ACE2, angiotensin-converting enzyme 2; MODS, multiple organ dysfunction syndrome.

### 4.1 Common mechanism: convergent pathways to the endothelial-immunothrombotic storm

Although the mechanisms of viral and bacterial infection differ, their pathological processes become highly convergent once the host response becomes dysregulated. This convergence is specifically manifested by: (1) the sustained amplification of SIRS; (2) widespread endothelial cell activation and injury (endotheliopathy); (3) immunothrombosis driven by coagulation imbalance; culminating in the development of MODS (1, 8, 67). This shared pathological trajectory indicates that, regardless of the initial insult, a dysregulated host response fundamentally operates around the core axis of inflammation–endothelium–coagulation.

Furthermore, following the peak inflammatory phase, patients with both viral and bacterial sepsis can enter a stage of immunosuppression. This stage is characterized by significant lymphopenia and T-cell functional exhaustion, which increases the risk of secondary infections and clinical deterioration (68). Throughout this evolution, key immunopathological events, such as NETosis, complement activation, and cytokine storm, have been documented in both sepsis types. Although differences may exist in their intensity and molecular profiles, these events collectively promote inflammatory dissemination, endothelial damage, and tissue injury (69).

### 4.2 Key differences

#### 4.2.1 Differences in initiating factors

The first key divergence between bacterial and viral sepsis originates from their distinct mechanisms of pathogen recognition. Bacteria are primarily recognized by membrane-bound receptors such as TLR2 and TLR4. This recognition rapidly triggers a robust, NF-κB-centered pro-inflammatory response, representing a centralized initiation mode dominated by circulating immune cells (10). In contrast, viral nucleic acids and related molecules can be sensed simultaneously within immune cells, epithelial cells, and endothelial cells via intracellular pathways including TLR3/7/8/9, RIG-I/MDA5, or the cGAS–STING pathway. This leads to a decentralized, pan-cellular initiation and generates an antiviral response that is more dependent on the timing and dynamics of interferon signaling (13, 15). Meanwhile, many viruses can directly infect and damage the endothelium. This results in primary endothelial injury early in the course of viral sepsis. Conversely, endothelial injury in bacterial sepsis more commonly occurs as a secondary process driven by bacterial toxins, complement activation, and high levels of inflammatory mediators (70). These fundamental differences at the initiation stage dictate divergent early inflammatory kinetics, endothelial response patterns, and the subsequent direction of immune-coagulation coupling (71).

#### 4.2.2 Systemic differences in coagulation activation patterns

Although both viral and bacterial sepsis can develop immunodriven coagulopathy, they exhibit highly divergent patterns in the kinetics, spatial distribution, and terminal phenotypes of coagulation activation. Viral sepsis, typified by COVID-19, more characteristically presents as a sustained hypercoagulable state. Marked elevations in D-dimer and fibrinogen are observed, while platelet counts typically remain normal or are only mildly decreased (72, 73). Pathologically, significant endotheliitis, excessive NET formation, and PAI-1 mediated fibrinolysis inhibition collectively skew coagulation activation toward a focal immunothrombotic pathway. This process is particularly concentrated within the pulmonary microcirculation (74, 75). Autopsy and imaging studies further indicate that the incidence of *in situ* pulmonary thrombosis and pulmonary embolism in COVID-19 exceeds that in typical bacterial sepsis. This pattern reflects a coagulation abnormality stemming primarily from a local imbalance between procoagulant and antifibrinolytic forces in the setting of virus-associated endothelial injury, rather than from systemic consumption of coagulation factors (6, 76).

In contrast, classical bacterial sepsis more frequently follows a trajectory toward consumptive coagulopathy, or disseminated intravascular coagulation (DIC) (77). Driven by sustained high levels of inflammatory mediators, tissue factor activity, and complement-coagulation cross-amplification, coagulation factors and platelets undergo rapid and massive consumption. This manifests as prolonged clotting times, significant thrombocytopenia, increased bleeding risk, and diffuse microthrombotic deposition (78, 79). The resultant coagulopathy in bacterial sepsis is thus characterized predominantly by systemic consumption and coagulant failure. This stands in stark contrast to the viral sepsis pattern, which centers on focal hypercoagulability and *in situ* thrombus formation (80). Therefore, the key distinction in coagulopathy between the two sepsis types lies not in the mere presence of thrombi, but in the spatial bias of activation (localized versus systemic), the kinetic pattern (sustained hypercoagulability versus rapid consumption), and the consequent laboratory and clinical phenotypes.

#### 4.2.3 Differences in endothelial injury patterns

Endothelial injury in viral sepsis follows a distinct upstream trigger chain that markedly differs from that in bacterial sepsis. In viral infections typified by COVID-19, the binding of the virus to angiotensin-converting enzyme 2 (ACE2) triggers receptor internalization. This leads to a concurrent downregulation of ACE2 surface expression and a reduction in its membrane-bound carboxypeptidase activity (81). This change not only compromises ACE2’s core enzymatic functions, including peptide metabolism, bradykinin degradation, inflammation modulation, and nitric oxide (NO) homeostasis, but also disrupts the balance between multiple, normally counter-regulatory signaling axes. This disruption constitutes a key mechanistic foundation for the primary endotheliopathy characteristic of viral sepsis (82).

First, ACE2 downregulation impedes the metabolic conversion of angiotensin (Ang) II to Ang-(1–7), leading to the sustained local accumulation of Ang II (83, 84). Animal studies confirm that high Ang II levels, acting via the AT1 receptor, drive NF-κB activation, NADPH oxidase-mediated oxidative stress, and the upregulation of adhesion molecules and tissue factor. This rapidly shifts the endothelial phenotype toward a proinflammatory and procoagulant state (85, 86). Concurrently, the dominant Ang II signaling suppresses endothelial nitric oxide synthase (eNOS) activity and reduces NO production. This impairs microvascular dilation, increases perfusion resistance, and lays the groundwork for subsequent permeability disruption and microcirculatory dysfunction (87).

ACE2 serves as the primary enzyme for degrading des-Arg^9^-bradykinin (DABK). Its downregulation reduces DABK clearance and potentiates signaling through the B1 receptor (88). This signaling axis induces cytoskeletal remodeling, disrupts intercellular junctions, significantly increases vascular permeability, and promotes neutrophil recruitment to lung tissue. It is thus a key driver of the focal edema and permeability imbalance observed early in viral sepsis (89). Consequently, blocking this axis is considered a potential strategy to interrupt this pathological cascade, offering a novel therapeutic target for COVID-19 (90).

Simultaneously, reduced generation of Ang-(1–7) diminishes Mas receptor-mediated anti-inflammatory, antioxidant, and antifibrinolytic pathways. This overall weakening of the protective regulatory axis leaves it unable to counteract the propagation of inflammatory and procoagulant signals (91, 92). The cascade initiated by receptor binding and internalization, the resultant ACE2 downregulation and enzymatic imbalance, and the concurrent weakening of protective axes alongside the strengthening of injurious ones collectively shape the pathology of viral sepsis. This process is characterized by early primary endothelial injury, manifesting as significantly increased permeability, amplified inflammation, and a predisposition toward focal immunothrombosis.

In contrast, endothelial injury in bacterial sepsis is primarily driven by bacterial toxins, complement activation, and a high inflammatory burden. It represents a form of secondary injury that does not involve the early signal reprogramming caused by ACE2 downregulation and internalization (93). Therefore, the two sepsis types differ fundamentally in their initiating mechanisms and progression trajectories. This distinction makes viral sepsis more prone to developing a coupled phenotype of permeability dysregulation and immunothrombosis.

#### 4.2.4 Changes in immune cell subsets

Viral sepsis exhibits immune cell subset alterations that are characterized by an earlier onset, greater severity, and longer duration, particularly within the T cell system. Multiple studies have shown that patients with COVID-19 exhibit significant reductions in CD4^+^ and CD8^+^ T cell counts early in the disease course. This is accompanied by the upregulation of inhibitory receptors such as PD-1and TIM-3, along with a decline in effector function. This T cell profile is closely linked to persistent antigenic stimulation and a type I interferon-dominated immune milieu, indicating that viral infection can induce T cell exhaustion and thereby weaken antiviral immunity (94–96). In contrast, lymphopenia in bacterial sepsis more commonly stems from acute inflammatory burden, apoptosis, or tissue redistribution, and its depth and persistence are generally less pronounced than in viral sepsis (95).

Moreover, viral sepsis is more prone to inducing an aberrant reshaping of adaptive immunity. This includes, in a subset of patients, the generation of autoantibodies such as anti-interferon antibodies, anti-phospholipid antibodies, or anti-ACE2 antibodies. These immune deviations not only impair antiviral defenses but can also promote vascular inflammation and immunothrombosis (97, 98). While bacterial sepsis similarly induces immunosuppression, it less frequently manifests this particular pattern of autoantibody-mediated immune dysregulation.

#### 4.2.5 Organ involvement patterns

Viral and bacterial sepsis exhibit marked divergences in the primary sites of organ involvement, their progression pathways, and the underlying structure of microcirculatory injury. As a disease caused by a respiratory virus, COVID-19 initially targets tissues with high expression of its viral receptor, particularly the pulmonary microvasculature and alveolar epithelium. This results in respiratory system injury characterized by acute respiratory distress, impaired gas exchange, and *in situ* immunothrombosis (6, 99). Subsequent endotheliitis, increased microvascular permeability, and focal immunothrombosis can then extend to affect organs such as the kidneys, heart, and central nervous system. This defines a multiorgan dysfunction pathway predominantly mediated by microcirculatory disturbances (29, 100). In contrast, the target organs in bacterial sepsis are more dependent on the primary site of infection (e.g., abdomen, pleural space, or urinary tract). The resulting organ injury is typically driven by a combination of systemic inflammatory burden, endotoxin exposure, complement terminal complex deposition, and diffuse microthrombosis, resulting in a broader and more variable spectrum of multiorgan involvement (101, 102).

Furthermore, the mechanisms of organ injury differ distinctly. In viral sepsis, renal involvement tends to be driven more by endotheliitis, focal thrombosis, and potential direct viral infection of cells. In contrast, renal injury in bacterial sepsis more frequently results from the combined effects of shock-induced hypoperfusion, microcirculatory collapse, and inflammatory tubular damage (29, 70). Similarly, myocardial involvement in COVID-19 is more closely linked to immune-mediated inflammation and microvascular pathology. Bacterial sepsis, however, predominantly leads to myocardial dysfunction through mechanisms involving myocardial depressant factors, metabolic derangements, and perfusion deficits (103).

### 4.3 Heterogeneous mechanisms of endothelial injury and immunothrombosis in different viral sepsis syndromes

Different viruses can perturb the endothelial barrier and trigger immunothrombosis through diverse upstream mechanisms, resulting in significantly heterogeneous pathogenic patterns (**Table 2**). Influenza virus is characterized by the acute amplification of innate immunity. Extensive NET release directly damages the pulmonary microvascular endothelium while concurrently promoting contact pathway coagulation activation. This makes focal immunothrombosis a primary pathological feature (104). Dengue virus primarily relies on nonstructural protein 1 (NS1)-mediated glycocalyx disruption to rapidly destabilize the endothelial barrier. This manifests as increased vascular permeability and plasma leakage (105). The core pathogenesis of hantavirus lies in its direct infection of microvascular endothelial cells. By disrupting the cytoskeleton and tight junctions, it causes a rapid and dramatic increase in capillary permeability. However, the associated inflammatory response and coagulation activation are typically more limited in intensity compared to other viral syndromes (106).

**Table 2:** Comparison of key characteristics of viral sepsis syndromes caused by different viruses.

Virus type	Characteristics of endothelial injury	Immune response profile	Coagulation/Immunothrombosis features	Major clinical manifestations
SARS-CoV-2 (81, 97)	ACE2 downregulation disrupts the RAAS/KKS balance, accompanied by multi-axial endothelial injury involving complement, platelets, and NETs.	Delayed interferon response with concurrent inflammatory amplification and immunosuppression.	Enhanced contact activation of FXII, increased NET formation, and inhibited fibrinolysis, resulting in an overall hypercoagulable state.	ARDS, pulmonary microthrombosis, hypercoagulable state, MODS.
Influenza Virus (104)	Predominantly inflammation-mediated secondary endothelial injury, with NETs causing further damage to the pulmonary microvascular endothelium.	Rapid and robust activation of innate immunity, with prominent neutrophil activation.	NETs promote activation of the intrinsic coagulation pathway, facilitating the formation of focal immunothrombi.	ARDS, pulmonary microthrombosis.
Dengue Virus (105)	NS1 disrupts the endothelial glycocalyx, leading to rapid barrier destabilization and significantly increased vascular permeability.	Concurrent interferon response and immune evasion, with imbalance in inflammation and permeability regulation.	Mild coagulation abnormalities; the disease course is primarily characterized by capillary leakage.	Severe capillary leak, hypovolemic shock.
Hantavirus (106)	Direct infection of microvascular endothelial cells, damaging the cytoskeleton and tight junctions, leading to markedly increased permeability.	Strong early interferon response with a moderate degree of inflammation.	Relatively mild coagulation activation, dominated by a permeability storm.	Pulmonary edema, renal injury, HFRS.

ACE2, angiotensin-converting enzyme 2; ARDS, acute respiratory distress syndrome; FXII, coagulation factor XII; IFN, interferon; KKS, kallikrein–kinin system; MODS, multiple organ dysfunction syndrome; NETs, neutrophil extracellular traps; NS1, nonstructural protein 1; PAI-1, plasminogen activator inhibitor-1; RAAS, renin–angiotensin–aldosterone system.

In contrast to the patterns described above, the endothelial injury pathway induced by SARS-CoV-2 is more complex and multifaceted. It involves not only disruption of the receptor-enzyme system via ACE2 downregulation and renin-angiotensin-aldosterone system (RAAS) imbalance, but also multi-axis amplification effects including complement activation, platelet activation, and synergistic NETs activity. These processes cause inflammation, coagulation, and permeability alterations to become concurrently and tightly coupled (81, 97). This composite injury pattern stands in complementary contrast to the inflammation-NET-dominant mechanism of influenza, the barrier-disruptive mechanism of dengue, and the direct infection mechanism of hantavirus. This comparison further underscores the high degree of biological diversity inherent in viral sepsis syndromes.

### 4.4 Refining the “storm” conceptual framework

Despite significant differences in tissue tropism, injurious molecules, and immunomodulatory strategies among viruses, their pathological processes ultimately converge at the endothelium as a central hub. For instance, dengue virus disrupts the endothelial glycocalyx via nonstructural protein 1, hantavirus causes a precipitous increase in permeability through direct endothelial infection, and influenza virus drives NETosis and focal procoagulant responses. Although their upstream triggering mechanisms differ, they share the common terminal outcomes of endothelial barrier disruption, amplified local inflammation, and promotion of immunothrombosis. Thus, the endothelium serves not only as an anatomical target for viral pathogenesis but also as the critical pathological nexus integrating inflammation, complement activation, coagulation, and microcirculatory dysfunction. This allows the heterogeneous injury patterns induced by diverse viruses to achieve a unified pathological integration at the endothelial level.

The emergence of COVID-19 has further solidified this conceptual framework. SARS-CoV-2-induced ACE2 downregulation and multi-axis homeostatic imbalance establish primary endotheliopathy as the earliest and most decisive starting point in the disease course. Subsequently, highly active NETosis, aberrant platelet activation, and pronounced fibrinolysis inhibition on the damaged endothelial surface continuously amplify microthrombotic deposition. Concurrently, dysregulated interferon signaling, T cell exhaustion, and autoantibody production in some patients further weaken negative regulatory mechanisms. This combination facilitates the synchronous spread of microcirculatory impairment to organs such as the lungs, kidneys, heart, and brain. In summary, although different viruses initiate the pathological cascade through distinct mechanisms, they ultimately drive organ failure via the common axis of endothelial injury, immunothrombosis, and microcirculatory collapse. This constitutes the shared foundation of the endothelial-immunothrombotic storm in viral sepsis.

## 5 Clinical implications and translational research

A deeper understanding of the mechanisms underlying the endothelial-immunothrombotic storm holds significant clinical and translational importance for the diagnosis, prognostic assessment, and therapeutic strategy development in viral sepsis, particularly in COVID-19.

### 5.1 Diagnosis and prognosis

Current diagnosis of sepsis primarily relies on clinical criteria and organ function scores, lacking specific biomarkers that can accurately reflect the activity state and severity of the endothelial-immunothrombotic storm. Future research should focus on exploring multi-dimensional, multi-pathway biomarker panels to more comprehensively capture pathological features such as immune activation, endothelial dysfunction, coagulation abnormalities, and immune dysregulation, thereby optimizing the early identification and precise phenotyping of sepsis.

Firstly, endothelial injury markers, such as sTM, Ang-2, soluble ICAM-1, soluble VCAM-1,vWF, and the ratio of vWF to its cleaving protease ADAMTS13, correspond to the most upstream node of the storm. They reflect glycocalyx disruption, altered barrier permeability, and the remodeling of the endothelium toward a proinflammatory and procoagulant phenotype, constituting core indicators of the storm’s initiation phase (107–109). Secondly, immune activation markers, including IL-6, IL-1RA, soluble CD14 (sCD14), sCD163, interferon-γ-inducible protein 10, and TNF-α, capture the amplified feedback within innate immune pathways involving neutrophils, monocytes, and macrophages. These markers link the inflammatory burden to the degree of endothelial injury and serve as a key source of information during the immune amplification stage (95). For example, a retrospective study showed that levels of IL-6, IL-1β, and TNF-α were significantly elevated in patients with severe COVID-19, while IFN-γ levels were markedly downregulated compared to those with mild disease (110). Thirdly, coagulation and fibrinolysis system indicators—such as D-dimer, thrombin-antithrombin complex, prothrombin fragment F1 + 2, PAI-1, and fibrin monomers—represent the extent to which the storm network progresses toward an immunothrombotic response. They reveal terminal effector programs including thrombin generation, fibrin deposition, and fibrinolysis inhibition (111). Finally, NET-related markers, such as cell-free DNA, myeloperoxidase-DNA complexes, and citrullinated histone H3, reside at the convergence point of the storm. They reflect both the immunopathological response of granulocytes and directly participate in coagulation factor activation and microvascular occlusion, acting as crucial amplifiers within the storm’s positive feedback loop (112, 113).

In addition, the advancement of multi-omics technologies has enabled the molecular landscape of the endothelial-immunothrombotic storm to be deconstructed at a systems level. Techniques such as transcriptomics, proteomics, metabolomics, and single-cell sequencing allow for the systematic analysis of the molecular modules, perturbed signaling pathways, and cellular lineage reprogramming associated with endothelial activation, inflammatory amplification, immune exhaustion, and immunothrombosis. This reveals the mechanistic hallmarks of different storm stages and the heterogeneity among patients (59, 114). Several studies indicate that in both COVID-19 and non-COVID-19 sepsis, non-survivors consistently exhibit sustained gene expression dysregulation. This includes persistent suppression of T cell signaling alongside continuous overactivation of innate immune and metabolism-related pathways, constituting an immunodysfunctional phenotype that is difficult to reverse (94, 96, 110). These conserved molecular imbalances across diseases suggest that high-dimensional mechanistic information is more indicative of critical disease junctures than traditional clinical parameters.

However, multi-omics data are challenging to apply directly in clinical settings. There is an urgent need to distill this information into biomarker panels that can be tested at the bedside. Given that individual biomarkers often exhibit non-linear and interdependent dynamic changes during the disease course, the research focus should shift from single indicators to mechanism-based combinatorial signatures. A multi-dimensional biomarker panel constructed around the key nodes of the storm (**Table 3**) holds promise for dynamically assessing disease status, identifying optimal therapeutic windows, and enabling more precise, individualized patient management.

**Table 3:** Viral sepsis–related biomarkers and their clinical significance.

Category	Key biomarkers	Pathophysiological and clinical significance	Reference(s)
Endothelial Activation /Injury Markers	Soluble thrombomodulin, angiopoietin-2, soluble ICAM-1/VCAM-1, vWF, vWF multimers, ADAMTS13 activity	Reflect endothelial barrier dysfunction, overexpression of adhesion molecules, and a procoagulant tendency, indicating increased microvascular permeability and association with microvascular pathology	(107–109)
Cytokine Storm and Immune Activation	IL-6, TNF-α, IL-8, CXCL10, soluble CD14, soluble CD163	Indicate a proinflammatory cytokine storm and myeloid cell activation status, associated with disease progression and organ dysfunction	(17, 95, 110)
Immune Dysregulation /Exhaustion	Low HLA-DR expression (on monocytes), PD-1, TIM-3, LAG-3	Represent adaptive immune exhaustion and impaired antigen presentation, suggesting susceptibility to secondary infections and immune paralysis	(25, 49, 68, 94)
Coagulation Disorders and Fibrinolysis Inhibition	D-dimer, TAT complex, prothrombin fragment F1 + 2, PAI-1, fibrin monomers	Indicate activation of the coagulation cascade, suppression of the fibrinolytic system, and increased microvascular thrombotic burden, closely associated with the risk of developing MODS	(97, 111, 118)
NETs-Related Markers	Cell-free DNA (cfDNA), MPO–DNA complexes, citrullinated histone H3	Reflect active formation of neutrophil extracellular traps (NETs), suggesting a role in promoting immunothrombosis	(57, 60, 97)
Complement System Activation Markers	C3a, C5a, soluble C5b-9	Indicate excessive activation of the complement cascade, amplifying inflammatory and coagulation responses, and promoting endothelial dysfunction	(36, 44, 45, 90)
Autoimmunity-Related Markers	Antiphospholipid antibodies, anti-interferon, antibodies, anti-ACE2 antibodies	Reflect a breakdown of immune tolerance and potential autoimmune responses, possibly contributing to microvascular thrombosis and sustained inflammation	(51, 52, 108)

ICAM-1, intercellular adhesion molecule-1; VCAM-1, vascular cell adhesion molecule-1; vWF, von Willebrand factor; ADAMTS13, a disintegrin and metalloproteinase with thrombospondin motifs 13; IL-6, interleukin-6; IL-1RA, interleukin-1 receptor antagonist; TNF-α, tumor necrosis factor-alpha; HLA-DR, human leukocyte antigen–DR; PD-1, programmed cell death protein 1; TIM-3, T-cell immunoglobulin and mucin-domain containing-3; LAG-3, lymphocyte activation gene 3; TAT complex, thrombin-antithrombin complex; F1 + 2 fragment, prothrombin fragment 1 + 2; PAI-1, plasminogen activator inhibitor-1; cfDNA, cell-free DNA; MPO-DNA, myeloperoxidase-DNA complex; CitH3, citrullinated histone H3; C3a and C5a, complement activation products; sC5b-9, soluble terminal complement complex; ACE2, angiotensin-converting enzyme 2; MODS, multiple organ dysfunction syndrome.

### 5.2 Therapeutic strategies

The endothelial-immunothrombotic storm requires a multimodal, pathway-targeted approach that integrates antiviral therapy, immune modulation, inflammation control, correction of coagulopathy, and endothelial protection. To facilitate a holistic understanding of this framework, **Table 4** summarizes the major pathological components and their corresponding potential interventions in a structured format.

**Table 4:** Core pathological processes and corresponding intervention strategies in viral sepsis.

Pathological process	Key mechanistic features	Potential targeted therapeutic strategies	Reference(s)
Pathogen Recognition and Invasion	SARS-CoV-2 enters endothelial cells via ACE2, activates PRRs (TLR3/7/9, RIG-I), inducing type I/III interferons and early antiviral responses	Antiviral agents (Paxlovid, remdesivir)	(14, 81, 115)
Immune Activation and Immune Imbalance	Excessive release of proinflammatory cytokines (IL-6, TNF-α, etc.) driving the cytokine storm, along with T-cell exhaustion (PD-1↑, HLA-DR↓), CD8^+^ dysfunction, and even autoantibody production	IL-6/IL-1 antagonists, JAK inhibitors, Treg-enhancing therapies	(17, 96, 125)
Endothelial Dysfunction	Viral infection or cytokine-mediated endothelial activation induces upregulation of ICAM-1, vWF, and PAI-1, leading to barrier disruption and a procoagulant phenotype	ACEIs, ARBs, APC	(73, 82, 134)
Amplification via NETs and Complement	NETosis, C5a and complement cascade activation, driving a coagulation–inflammation amplification loop	Anti-C5a antibodies, DNase I, PAD4 inhibitors	(58, 88, 97)
Coagulation Abnormalities and Thrombosis	Elevated PAI-1 and D-dimer, suppressed fibrinolysis, increased microvascular thrombotic burden	Heparin, recombinant thrombomodulin, DOACs	(59, 73, 113)
End-Organ Injury and MODS	Microvascular thrombosis causes tissue hypoperfusion and hypoxia, ultimately progressing to ARDS, acute kidney injury, myocardial injury, and MODS	Early organ support, individualized life support strategies	(59, 70, 90)

ACE2, angiotensin-converting enzyme 2; PRRs, pattern recognition receptors; TLRs, Toll-like receptors; RIG-I, retinoic acid-inducible gene I; IFN-I/III, type I/III interferons; IL-6, interleukin-6; TNF-α, tumor necrosis factor-alpha; PD-1, programmed cell death protein 1; HLA-DR, human leukocyte antigen–DR; CD8^+^, cluster of differentiation 8 positive T cell; ICAM-1, intercellular adhesion molecule-1; vWF, von Willebrand factor; PAI-1, plasminogen activator inhibitor-1; APC, activated protein C; NETs, neutrophil extracellular traps; C5a, complement component 5a; PAD4, peptidylarginine deiminase 4; D-dimer, fibrin degradation product D-dimer; DOACs, direct oral anticoagulants; ARDS, acute respiratory distress syndrome; MODS, multiple organ dysfunction syndrome.

#### 5.2.1 Antiviral therapy

Within this pathological network, viral replication represents the earliest and most essential upstream driver of the endothelial–immune thrombotic storm. Effective antiviral therapy can reduce viral load, thereby limiting endothelial infection, attenuating inflammatory stimuli, and dampening downstream immune and coagulation cascade activation. In viral sepsis exemplified by COVID-19, small-molecule antivirals such as nirmatrelvir–ritonavir and remdesivir have demonstrated the ability to reduce disease progression when administered during the early viral-replicative phase (115, 116). In other forms of viral sepsis, such as dengue, influenza, and enteroviral infections, early initiation of pathogen-specific antiviral agents, when available, is likewise crucial for preventing subsequent amplification of immune and coagulation responses. It is important to note, however, that viral rebound may still occur despite early antiviral administration, as documented in studies reporting SARS-CoV-2 recurrence following Nirmatrelvir–Ritonavir therapy (117).

#### 5.2.2 Anti-inflammatory/immunomodulatory therapy

Glucocorticoids: Within the network characterized by endothelial activation, immune amplification, and immunothrombosis, excessive inflammation is a central driver of microcirculatory dysfunction and tissue injury (118). Consequently, anti-inflammatory and immunomodulatory strategies are essential in the management of viral sepsis. These strategies aim to suppress cytokine cascades, restore immune homeostasis, and reduce endothelial injury and thrombogenesis mediated by inflammation. Among available interventions, glucocorticoids represent one of the most well-established immunomodulatory therapies supported by robust clinical evidence (119). In viral sepsis caused by COVID-19, dexamethasone has been shown to reduce mortality in patients requiring supplemental oxygen or mechanical ventilation. Its therapeutic effects arise not only from dampening systemic inflammation but also from reversing transcriptional dysregulation in monocytes and indirectly mitigating abnormal interactions across immune, coagulation, and endothelial pathways (120).
Targeted modulation of the IL-1 and IL-6 axis: In patients with a hyperinflammatory phenotype or clinical features consistent with a cytokine storm, therapies directed at IL-1 and IL-6, two central nodes within the inflammatory cascade, provide an important means of limiting immune amplification. IL-6 is a key mediator of systemic inflammation and the acute-phase response. Its receptor antagonists tocilizumab and sarilumab have shown potential benefit in selected patients with severe COVID-19, particularly in hyperinflammatory subgroups characterized by markedly elevated C-reactive protein and IL-6 concentrations (121, 122). IL-1 plays a crucial role during the early stages of innate immune activation, cytokine production, and propagation of the inflammatory cascade. Anakinra, an IL-1 receptor antagonist, can attenuate early inflammatory amplification and endothelial injury in patients with macrophage activation syndrome–like features or other high-inflammation states. Signals suggesting potential improvement in clinical course or survival have been observed in studies involving both COVID-19 and bacterial sepsis (123, 124). Taken together, these interventions illustrate that blockade of key inflammatory hubs may mitigate immune-mediated tissue injury and microcirculatory dysfunction.
JAK inhibitors: Broad-spectrum cytokine blockade can also be achieved with Janus kinase inhibitors, which suppress the JAK–STAT signaling pathway and thereby interrupt the activity of multiple inflammatory mediators, including interleukin 6 and interferon gamma. Baricitinib, the most widely studied agent in this class, has demonstrated evidence of improved clinical outcomes in severe COVID-19, both as monotherapy and in combination with remdesivir (125). In addition, ruxolitinib can markedly reduce the release of proinflammatory cytokines by inhibiting JAK–STAT5 signaling, providing a mechanistic basis for its potential immunomodulatory role in correcting inflammation dysregulation in sepsis (126).
Complement inhibitors: The complement system resides at the convergence of inflammatory amplification, coagulation activation, and endothelial injury, serving as a crucial upstream hub that drives the immune-endothelial-coagulation storm. Consequently, complement inhibitors are increasingly regarded as a key strategic intervention for disrupting this cascade. Various targeted complement therapeutics, including anti-C5a monoclonal antibodies, anti-C5 monoclonal antibodies, and C3 inhibitors, have demonstrated promising efficacy in clinical studies for both COVID-19 and sepsis. These findings support the notion that complement blockade may improve patient outcomes by mitigating complement-mediated endothelial damage and microvascular thrombosis (127–129). Notably, a multicenter randomized controlled trial involving 368 patients demonstrated that the anti-C5a antibody vilobelimab significantly reduced 28-day mortality and improved survival outcomes in critically ill COVID-19 patients requiring invasive mechanical ventilation (127).
Other exploratory approaches: Several additional therapeutic strategies directed at more specific mechanisms are also under continuous development. For example, supplementation with IFN-α or IFN-β to restore early antiviral responses has shown potential clinical benefit. A multicenter retrospective study reported that early IFN-α administration was associated with lower in-hospital mortality, and a randomized controlled trial (RCT) demonstrated that inhaled IFN-β significantly improved clinical outcomes (130, 131). In addition, removal of NETs is considered an important approach for interrupting immunothrombosis. Preclinical studies have shown that local delivery of DNase I can continuously degrade pulmonary NETs, alleviate NET-mediated inflammation and tissue injury, and markedly improve pathological features in models of acute lung injury (132). These exploratory therapies provide an important foundation for the development of future precision immunomodulatory strategies.

#### 5.2.3 Anticoagulation and regulation of immunothrombosis

In both viral sepsis and bacterial sepsis, microvascular immunothrombosis is a central pathological process that contributes to impaired organ perfusion and tissue injury. Anticoagulation therefore serves not only to prevent thromboembolism but also to suppress immunothrombus formation, improve microcirculatory perfusion, and mitigate endothelial injury. Unfractionated heparin and low molecular weight heparin have been widely used for thromboprophylaxis and treatment in COVID-19 because they combine anticoagulant, anti-inflammatory, and potential endothelial-protective effects (132). Nevertheless, the choice between therapeutic-dose and prophylactic-dose heparin in critically ill patients with COVID-19 remains controversial. Clinical decisions require an integrated assessment of thrombotic risk, bleeding tendency, inflammatory burden, and coagulation profile, with individualized dosing strategies. In classical bacterial sepsis, when sepsis-induced coagulopathy or DIC is present, prophylactic-dose heparin is typically preferred. Initiation of anticoagulation is generally considered only when there is no significant bleeding risk[ (133, 134). Compared with viral sepsis, which is characterized by a predominant hypercoagulable state and *in situ* thrombosis, anticoagulation in bacterial sepsis is more focused on preventing ongoing consumption of coagulation factors and suppressing diffuse microvascular clot deposition.

Furthermore, direct oral anticoagulants (DOACs) demonstrate potential utility in long-term thromboprophylaxis following discharge for patients with COVID-19-associated thromboembolism. However, evidence regarding their safety and efficacy during the acute in-hospital phase, particularly in critically ill patients, remains limited. Therefore, DOACs have not been recommended as first-line anticoagulant therapy in this setting (135, 136). Consequently, with the deepening understanding of immunothrombosis mechanisms, anticoagulation management is gradually shifting from a traditional empiric dosing model toward a precision framework guided by underlying pathology. In the future, dynamically tailoring anticoagulant intensity and timing based on multi-dimensional biomarkers, such as D-dimer levels, fibrinolysis inhibition markers, and thromboelastography parameters, could maximize antithrombotic benefits while minimizing bleeding risk. This approach would establish a more robust evidence base for individualized anticoagulant management.

#### 5.2.4 Endothelial protection and repair strategies

In the endothelial immune thrombotic storm, endothelial cells are not only the earliest targets of injury but also the central hub through which inflammation, coagulation, and microcirculatory dysfunction reinforce one another. Restoring endothelial barrier stability, suppressing the shift toward proinflammatory and procoagulant phenotypes, and maintaining microvascular perfusion are therefore critical therapeutic directions for interrupting this vicious cycle.

Recombinant human APC (rhAPC) and rrTM represent strategies aimed at improving endothelial function by restoring the endogenous anticoagulant and cytoprotective axis. Although early use of rhAPC failed in sepsis because of bleeding risk, growing mechanistic insight into the cytoprotective actions of APC and the development of safer APC analogues have renewed interest in its potential value in selected subgroups (137). In addition, rTM has been approved in Japan for sepsis-associated DIC, and it improves endothelial function by promoting APC generation, reducing thrombin activity, and alleviating endothelial inflammation. These mechanisms directly target the endothelial injury driven by imbalance of the anticoagulant system within the storm network (138, 139).

A second category of therapies aims to correct abnormalities in inflammation and vascular tone regulation. Angiotensin converting enzyme inhibitors and angiotensin receptor blockers could theoretically improve endothelial stability and oxidative stress by modulating dysregulated RAAS activity. However, clinical evidence in acute sepsis remains limited, and careful evaluation of their hemodynamic effects is required (140, 141). Statins, which exert pleiotropic effects such as increasing NO bioavailability, suppressing inflammatory responses, and stabilizing the vascular wall, provide biologically plausible benefits for improving microcirculatory perfusion and preserving endothelial integrity. Although some studies suggest potential adjunctive value in sepsis and COVID-19, their true clinical benefit and optimal use scenarios still require validation in higher-quality trials (142, 143).

#### 5.2.5 Supportive therapy

In the storm state characterized by ventilation–perfusion mismatch and microcirculatory dysfunction driven by endotheliitis and immunothrombosis, respiratory support should prioritize low tidal volume lung protective ventilation and prone positioning as foundational strategies. When conventional measures fail to maintain adequate oxygenation, venovenous extracorporeal membrane oxygenation may be considered for rescue therapy (144). For circulatory management, recent randomized trials have demonstrated that a restrictive fluid strategy combined with early and individualized initiation of vasoactive agents is safe and feasible in patients with septic shock who have completed initial resuscitation. These approaches help avoid excessive fluid overload (42, 145, 146). In cases complicated by acute kidney injury, large RCTs have shown that renal replacement therapy, when initiated according to appropriate indications, can maintain fluid, metabolic, and electrolyte homeostasis without increasing mortality risk, thereby supporting overall organ function (42). Importantly, implementation of supportive therapies must account for the impact of the storm state on organ function. For example, in patients with ARDS who have severe pulmonary inflammation and endothelial injury, lung protective strategies are even more essential to avoid ventilator associated lung injury (147).

#### 5.2.6 Exploration of novel therapies

The intrinsic coagulation pathway and its cross activation with the KKS have increasingly been recognized as key upstream drivers of the combined amplification of coagulation, inflammation, and vascular permeability in COVID-19 (148). SARS-CoV-2 induced NETosis, endothelial disruption, and exposure of negatively charged extracellular matrices together promote contact activation of FXII, which initiates the FXIIa–kallikrein–bradykinin cascade. This cascade not only drives thrombin generation and fibrin deposition but also results in excessive production of bradykinin and its metabolite DABK (57). Downregulation of ACE2 further impairs DABK clearance, and the bradykinin and B1–B2 receptor axis subsequently induces vasodilation, increased vascular permeability, and amplified inflammation, creating a self reinforcing pathological circuit across the coagulation, inflammatory, and kinin pathways (88).

Based on these mechanisms, the FXII–KKS axis has emerged as a mechanism-oriented therapeutic target in COVID-19. In the ICAT-COVID RCT (NCT04978051), the bradykinin pathway inhibitor icatibant suggested a potential benefit for improving recovery in patients with early hypoxemic pneumonia (149). Two additional completed studies (NCT05407597 and NCT05010876) are evaluating its effects on bradykinin-mediated vascular permeability and inflammatory regulation. Garadacimab, an FXIIa neutralizing monoclonal antibody that targets the upstream contact system, did not show a clear clinical benefit in a phase II trial in severe COVID-19 (NCT04409509). However, the study confirmed target engagement and safety, supporting the feasibility of upstream inhibition within the contact pathway (150). A recombinant C1 inhibitor with multi-axis inhibitory properties against FXIIa, kallikrein, and complement activation also failed to demonstrate clinical improvement in a RCT (NCT04414631) and was terminated early for futility (151). It is noteworthy that two additional completed studies (NCT04705831 and NCT04530136) also focus on the bradykinin axis, and their results may clarify the true clinical value of FXII–KKS targeted therapy in the regulation of COVID-19 related immunothrombosis.

In addition, several novel endothelial-targeted therapies are in development. These include approaches designed to regulate vascular permeability, such as agents targeting vascular endothelial cadherin, suppress endothelial adhesion molecule expression, reduce oxidative stress, and modulate endothelial pyroptosis (152–154). These emerging strategies illustrate a shift in endothelial protection from traditional anticoagulant or anti-inflammatory concepts toward mechanism-based, multi-axis intervention frameworks.

### 5.3 Challenges and future directions

Although understanding of the storm network composed of endothelial injury, immune amplification, and immunothrombosis continues to advance, its clinical translation remains limited by several factors. Patients with sepsis and COVID-19 exhibit substantial heterogeneity in immune phenotypes, disease trajectories, and responses to therapy. This heterogeneity makes traditional uniform treatment approaches inadequate for the diverse pathological drivers and necessitates a shift from empirical management toward precision interventions guided by molecular phenotyping and risk stratification. In addition, the absence of bedside tools capable of real time assessment of storm intensity and dynamic fluctuations hampers timely adjustment of immunomodulatory, anticoagulant, or endothelial protective therapies according to pathological rhythms. Determining how to balance immune suppression and immune overactivation, and how to define the optimal timing, dosage, and duration of anti-inflammatory, anticoagulant, and targeted treatments, remains one of the most critical yet unresolved challenges. Premature or overly aggressive intervention may increase the risk of secondary infection, whereas delayed intervention may miss the window for disease reversal.

COVID-19 has provided a unique opportunity to develop the storm model, but different viruses such as influenza virus, dengue virus, and Ebola virus have distinct tissue tropism, immune activation pathways, and endothelial injury patterns. Their corresponding storm phenotypes and driving mechanisms require dedicated investigation to establish a framework that is both broadly applicable and reflective of virus specific features. Increasing evidence also suggests that acute phase storms may contribute to long term sequelae, including post acute COVID-19 syndrome and post sepsis syndrome, through mechanisms such as persistent low grade inflammation, residual endothelial dysfunction, or autoimmune activation (155). The biological basis and modifiability of these residual effects remain inadequately understood and represent important priorities for future research.

## 6 Conclusions and perspectives

COVID-19, as a representative model of viral sepsis, has advanced the concept of a storm network composed of endothelial injury, immune amplification, and immunothrombosis into a mechanistically defined framework, establishing it as a central pathological pathway leading to organ failure. Compared with bacterial sepsis, viral sepsis demonstrates receptor dependent tissue tropism, interferon dysregulation driven immune dynamics, and microcirculatory dysfunction dominated by hypercoagulability and *in situ* immunothrombosis. These features underscore the upstream role of disrupted endothelial homeostasis in disease progression.

Future research should focus on developing multidimensional biomarker panels that capture storm activity to enable precise early risk stratification and dynamic disease monitoring. It is also essential to identify the subgroups most likely to benefit and the optimal therapeutic windows for anti-inflammatory, anticoagulant, immunomodulatory, and endothelial protective interventions. Pathways involving NETs, complement activation, and endothelial regulation represent promising therapeutic nodes that merit further investigation because of their potential relevance to both acute outcomes and long term sequelae.

Overall, elucidation of the mechanisms underlying the endothelial immune thrombotic storm has deepened understanding of viral sepsis and provided a theoretical foundation for improving the diagnosis and treatment of sepsis and other diseases characterized by inflammation and coagulation interplay. Transforming these mechanistic insights into accessible clinical tools represents one of the most critical challenges and opportunities for the future.
